# *Brucella* Infections in Camels and Abattoir Workers in the United Arab Emirates: One Health-Based Seroepidemiological and Molecular Insights

**DOI:** 10.1155/tbed/7748638

**Published:** 2025-09-19

**Authors:** Gobena Ameni, Aboma Zewude, Berecha Bayissa, Abdallah A. Albizreh, Naeema Alhosani, Meera Saeed Alkalbani, Mohamed Moustafa Abdelhalim, Assem Sobhi Abdelazim, Rafeek Aroul Koliyan, Kaltham Kayaf, Mervat Mari Al Nuaimat, Robert Barigye, Balázs Ádám, Mohamud Sheek-Hussein, Guangzhi Zhang, Yassir Mohammed Eltahir, Markos Tibbo

**Affiliations:** ^1^Department of Veterinary Medicine, College of Agriculture and Veterinary Medicine, United Arab Emirates University, Al Ain, Abu Dhabi, UAE; ^2^Vaccine Production and Drug Formulation Directorate, National Veterinary Institute, Debre Zeit, Ethiopia; ^3^Department of Geography, College of Humanities and Social Sciences, United Arab Emirates University, Al Ain, UAE; ^4^Extension Services and Animal Health Division, Abu Dhabi Agriculture and Food Safety Authority, Abu Dhabi, UAE; ^5^Animal Development and Health Department, Ministry of Climate Change and Environment, Dubai, UAE; ^6^Institute of Public Health, College of Medicine and Health Sciences, United Arab Emirates University, Al Ain, Abu Dhabi, UAE; ^7^Institute of Animal Science, Chinese Academy of Agricultural Sciences, Beijing, China; ^8^Subregional Office for the Gulf-Cooperation Council States and Yemen, Food and Agriculture Organization of the United Nations, Abu Dhabi, UAE

**Keywords:** abattoir workers, *Brucella* DNA, *Brucella* infection, *Brucella* species, dromedary camels, seroprevalence, zoonosis

## Abstract

Brucellosis remains a critical zoonotic disease affecting livestock productivity and human health, especially in regions where intensive livestock husbandry is practiced. In the United Arab Emirates (UAE), camels contribute economically and culturally through meat, milk, and racing, yet data on brucellosis in camels and its zoonotic risk remain limited. This cross-sectional study aimed to determine the seroprevalence and molecular detection of *Brucella* species in camels and abattoir workers in the Emirate of Abu Dhabi, UAE. A total of 356 camels from 102 herds, 368 slaughter camels, and 86 abattoir workers were included. Competitive enzyme-linked immunosorbent assay (cELISA) was used for antibody detection, while species-specific quantitative PCR (qPCR) targeted *Brucella abortus*, *Brucella melitensis*, and *Brucella ovis* in seropositive samples. Herd-level seroprevalence was 10.8% (95% CI: 5.5%–18.5%) and significantly associated with herd size (*p*  < 0.05). Animal seroprevalence was 5.9% (95% confidence level [CI]: 3.7%–8.9%) in field camels and 3.0% (95% CI: 1.51%–5.30%) in slaughtered camels. Seven abattoir workers (8.1%) were seropositive, with butchers at greater risk. By species, *B. ovis*, *B. abortus* and *B. melitensis* were detected in camels, while only *B. ovis* and *B. abortus* were in abattoir workers. These findings indicate ongoing zoonotic risks in abattoir settings and support integrated One Health surveillance and control strategies.

## 1. Introduction

Dromedary camels (*Camelus dromedarius*) are of significant cultural, economic, and ecological importance across the arid and semiarid regions of the Middle East, North Africa, and parts of Asia. Camels are deeply embedded in both traditional heritage and modern economic development in the United Arab Emirates (UAE). Their contributions span a range of services and products, including transportation, meat and milk production, racing, beauty contests, and ecotourism. With rising regional and international demand for camel milk and meat, camels have transitioned from being multipurpose animals in subsistence systems to being key contributors to commercial livestock production [1]. In recent years, the UAE has made substantial investments in modernizing camel husbandry systems and enhancing productivity, making camel farming a vital component of the national food security strategy.

This study complements the global analysis by Dadar et al. [2], which reported an overall camel brucellosis prevalence of 7.6% and emphasized regional disparities and diagnostic inconsistencies. Our findings reflect these concerns, particularly the impact of local practices and the importance of molecular confirmation to address potential serological underdetection.

Despite their reputation for resilience in harsh desert environments, camels are not immune to infectious diseases, particularly zoonoses such as brucellosis. Brucellosis, caused by bacteria of the genus *Brucella*, is a major public health and livestock production concern globally [3]. This disease leads to reproductive failure, such as abortion, infertility, and stillbirth, resulting in significant economic losses [4]. In camels, the commonly implicated species are *Brucella abortus* and *Brucella melitensis*, both of which are highly pathogenic to humans [5]. Infected camels may appear asymptomatic, further complicating disease detection and control efforts [6].

Transmission occurs through direct contact with infected animals or the consumption of contaminated animal products, particularly unpasteurized milk and undercooked meat [7]. Traditional and semi-intensive management systems in the UAE, where camels often cohabit with small ruminants such as goats and sheep, increase the risk of interspecies transmission [8]. Additionally, the limited use of personal protective equipment among those who handle animals contributes to the occupational risk of brucellosis in humans.

Of particular concern is the potential zoonotic spillover to high-risk groups such as abattoir workers, herders, and veterinarians. Brucellosis in humans typically presents as undulant fever with a wide range of clinical manifestations, including musculoskeletal pain, endocarditis, and neurological symptoms. Chronic infection can lead to prolonged illness and significant loss of productivity [9]. Several studies in the UAE and other Gulf countries have reported cases of human brucellosis linked to camel contact or consumption of raw camel milk [10–14].

Emerging evidence suggests a broader host range for *Brucella ovis*, which is traditionally associated with rams and is not considered zoonotic. A recent study reported its presence in camels, raising questions regarding its transmission dynamics and zoonotic potential. This is of particular concern in settings where camels are raised with sheep, which is the primary reservoir of *B. ovis*.

Despite the significance of this disease, comprehensive epidemiological data on brucellosis in camels remain sparse, especially in the Gulf region. Most available studies rely on serological methods, which, while useful for surveillance, do not differentiate between *Brucella* species and lack the ability to detect active infections or coinfections. Molecular diagnostic tools, such as quantitative PCR (qPCR), offer improved sensitivity and specificity for identifying and characterizing *Brucella* infections [15].

Previous meta-analyses have demonstrated substantial variability in camel brucellosis seroprevalence across regions, with pooled estimates ranging from 4.2% to 9.8%, highlighting the need for targeted surveillance in high-risk environments [16]. Additional evidence from other Gulf and North African countries underscores the zoonotic transmission potential and underreporting of camel brucellosis [17, 18]. Therefore, this study was designed to address critical knowledge gaps in the epidemiology of camel brucellosis in the UAE. Specifically, this study aimed to: (i) estimate the seroprevalence of *Brucella* infection in dromedary camels raised under field and slaughter conditions in the Emirate of Abu Dhabi, (ii) identify the presence of *Brucella abortus*, *Brucella melitensis*, and *Brucella ovis* using qPCR, and (iii) assess the zoonotic risk among abattoir workers occupationally exposed to camels. By combining serological screening with molecular confirmation, this study provides a robust assessment of the burden and risk pathways of camel brucellosis, thereby informing integrated One Health interventions to safeguard animal and public health.

## 2. Materials and Methods

### 2.1. Description of the Study Area

**Figure 1:**
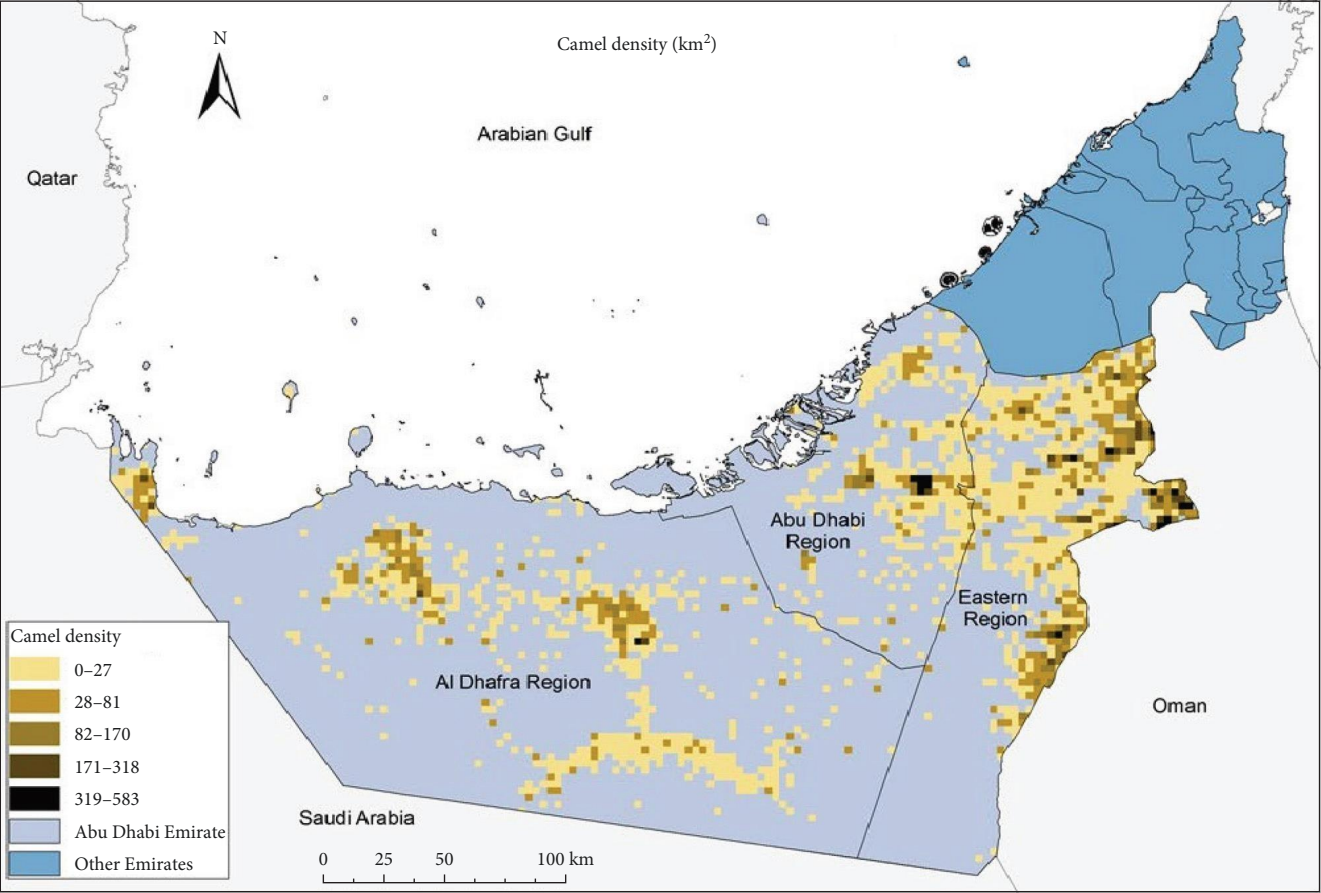
Density of camel population in the three regions of the Emirate of Abu Dhabi. The Emirate of Abu Dhabi is subdivided into three regions, namely the Abu Dhabi, the Eastern, and the Al Dhafra regions. The density of camel population is higher in the Abu Dhabi and Eastern regions as compared to the Al Dhafra region.

This study was conducted in the Emirate of Abu Dhabi in the UAE, encompassing three administrative regions (Figure 1): Abu Dhabi (Central), Al Ain (Eastern), and Al Dhafra (Western). These areas represent the major camel-producing zones in the country, characterized by an arid climate, limited annual rainfall (<100 mm), and pastoral or semi-intensive camel husbandry systems [19]. The Emirate of Abu Dhabi constitutes approximately 87% of the UAE's total land area [20]. The climate across the UAE, including the Emirate of Abu Dhabi, is classified as arid and is characterized by extremely hot summers (June to September). Coastal areas experience intense heat and humidity during this period, with temperatures reaching 46°C and a relative humidity approaching 100% [20]. In contrast, winter temperatures (December to March) range between 14 and 23°C, with a mean annual rainfall of approximately 78 mm, over 80% of which occurs during the winter months [20].

This study was conducted across all three administrative regions of the Emirate of Abu Dhabi, with additional sampling carried out at the Al Bawadi abattoir located in Al Ain City within the eastern region. Figure 1 illustrates the geographical distribution and population density of camels (per square kilometer) across the study area.

### 2.2. Study Design and Sampling Strategy

A cross-sectional study was conducted from May 2022 to March 2023 to determine the seroprevalence of *Brucella* spp. and assess associated risk factors in camels and abattoir workers. A multistage sampling method was used. First, herds were randomly selected from the Abu Dhabi Agriculture and Food Safety Authority (ADAFSA) registry, stratified by region. Second, camels were randomly sampled from each selected herd. The sample size for camels was determined using expected prevalence estimates from previous regional studies (5%–10%), with a 95% confidence level (CI) and 3% precision, resulting in 356 camels. For abattoir workers, convenience sampling was used among those who gave informed consent, with no statistical power calculation due to limited occupational groups. The sampling framework ensured representation from farms and traditional camel enclosures (*izbas*), which are defined as semi-structured or informal livestock holdings frequent in the UAE.

A total of 356 camels were sampled from field settings, including both farms and traditional enclosures, known as *izbas*. Blood was collected by veterinarians stationed at government veterinary clinics across three regions of the Emirate of Abu Dhabi. Each clinic sampled camels from farms and *izbas* in their catchment area.

In addition to field sampling, 368 camels used for slaughter at the Al Bawadi abattoir located in Al Ain were included in the study. These camels originate from various districts within the Emirate of Abu Dhabi, representing a broad geographic distribution. Furthermore, 86 of the 110 abattoir workers at Al Bawadi voluntarily consented to participate in this study. These individuals were primarily involved in activities that entailed direct contact with camel carcasses and biological material.

### 2.3. Sample Collection

Approximately 10 mL of blood was aseptically collected from the jugular vein of each camel and the antecubital vein of abattoir workers using sterile vacutainer tubes. The samples were transported on ice to the UAE University diagnostic laboratory. Sera were separated by centrifugation at 3000 rpm for 10 min and stored at −20°C until analysis.

### 2.4. Serological Testing

All serum samples were tested using a competitive enzyme-linked immunosorbent assay (cELISA) kit (ID Screen Brucellosis cELISA, IDvet, France), following the manufacturer's instructions. This assay detects antibodies against smooth lipopolysaccharide antigens of *Brucella* spp. Optical density (OD) was measured at 450 nm. A percentage inhibition (PI) ≥40% was considered positive [21]. The assay is validated for camelids with a reported sensitivity of 98% and specificity of 99% [22]. Positive and negative controls provided in the kit were used to validate test performance. No prior vaccination for *Brucella* spp. is implemented in UAE camels, eliminating potential cross-reactions due to immunization.

### 2.5. Molecular Detection of Brucella DNA

Molecular detection was performed only on seropositive samples to increase specificity. DNA was extracted using the NucleoSpin Tissue kit. Species-specific qPCR targeted *B. abortus*, *B. melitensis*, and *B. ovis*, with GAPDH used as an internal amplification control. Each run included positive controls (*Brucella* reference strains) and negative template controls. A sample was deemed positive if the Cq value was ≤40, consistent with prior studies [23]. Mixed infections were interpreted by the presence of distinct Cq values in more than one target assay. Amplification melt curve analysis was used to validate specific amplification. Species-specific qPCR assays targeting *Brucella abortus*, *Brucella melitensis*, and *Brucella ovis* were conducted using a CFX96 Real-Time PCR system (Bio-Rad, Hercules, CA, USA) with SYBR Green to monitor amplification. Primer sequences were obtained from previous studies [15, 23, 24]. Each sample was screened for the presence of all three *Brucella* species.

The *alkB* and *BMEI1162* genes, both located downstream of the IS711 insertion element, were targeted for detection of *B. abortus* and *B. melitensis*, respectively [15]. The *aroA* gene was targeted for *B. ovis* detection using specific primers [24]. Glyceraldehyde 3-phosphate dehydrogenase (GAPDH) was used as an internal control to assess DNA quality and amplification efficiency. Primer sequences used in this study are listed in Table 1.

PCR reactions were prepared using Maxima SYBR Green qPCR Master Mix (2×; Thermo Fisher Scientific, Waltham, MA, USA) in a final volume of 25 µL. Each reaction mixture contained 12.5 µL of master mix, 0.3 µM of each forward and reverse primer, 4 µL of template DNA, and nuclease-free water to adjust the final volume. The thermal cycling profile consisted of an initial incubation at 50°C for 2 min, followed by denaturation at 95°C for 10 min. Amplification was conducted over 40 cycles with denaturation at 95°C for 1 s and annealing/extension at 60°C for 30 s.

Amplification was performed using the CFX96 real-time system, with fluorescence captured using the FAM channel for target genes and the HEX channel for the internal control (GAPDH). A sample was considered positive for *Brucella* DNA when the quantification cycle (Cq or Ct) value was ≤40 [23, 24].

### 2.6. Data Management and Statistical Analysis

Statistical analyses were performed using the IBM SPSS Statistics version 29 (IBM Corp., Armonk, NY, USA). Descriptive statistics were used to calculate the seroprevalence of *Brucella* in camels and abattoir workers. Associations between seroprevalence and potential risk factors were assessed using the chi-squared (*χ*^2^) test, Fisher's exact test, or Wald test as appropriate for categorical variables. Binary logistic regression analysis was performed to estimate odds ratios (ORs) and 95% CIs to determine the strength and direction of the association between risk factors and seropositivity. Variables with a *p*-value < 0.20 in univariable analysis were included in the multivariable logistic regression model to control for potential confounding and identify independent predictors of *Brucella* seropositivity. Statistical significance was set at *p*  < 0.05.

### 2.7. Infographic Abstract of the M*ethodology*

**Figure 2:**
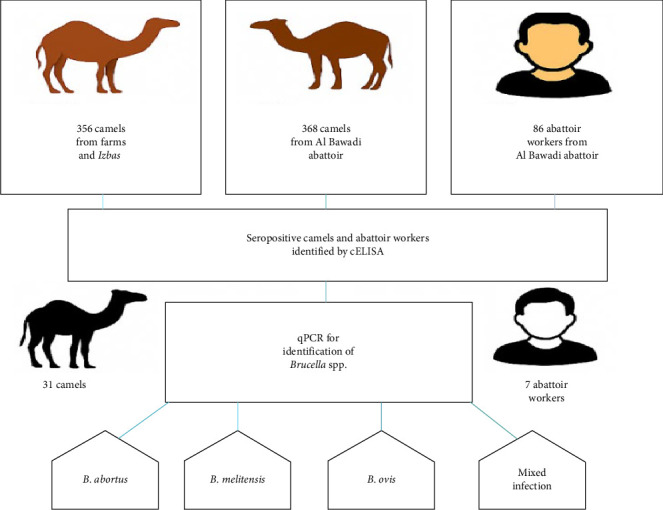
The infographic abstracts of the methodology used. A total of 356 camels from farms and *izbas*, 368 camels from Al Bawadi abattoir, and 86 abattoir workers were recruited for this study. All samples were screened for *Brucella* antibodies using competitive ELISA. Among them, 31 seropositive camels and seven seropositive abattoir workers were further tested using species-specific qPCR to detect *Brucella abortus*, *Brucella melitensis*, and *Brucella ovis*. The qPCR results revealed the presence of individual species as well as mixed infections in both camels and abattoir workers.

Figure 2 illustrates the methodological workflow of this study. Camel samples were collected from two sources: field holdings, including farms and traditional *izbas*, and the Al Bawadi abattoir. Human participants were exclusively recruited from among the abattoir workers at Al Bawadi. Serological testing was conducted using competitive ELISA (cELISA)to screen for *Brucella*-specific antibodies. Molecular detection was performed using qPCR. The qPCR assays were specifically designed to target three *Brucella* species of zoonotic and veterinary relevance: *B. abortus*, *B. melitensis*, and *B. ovis*.

## 3. Results

### 3.1. Herd-Level Seroprevalence of Brucella Infection and Associated Risk Factors

The herd-level seroprevalence of *Brucella* infection among camel populations in the Emirate of Abu Dhabi was found to be 10.8% (95% CI: 5.5–18.5), based on cELISA testing (Table 2). Statistical analysis indicated a significant association between seropositivity and herd size (*χ*^2^ = 7.73; *p*  < 0.05), suggesting that larger herds may have an increased risk of *Brucella* transmission, potentially due to more frequent animal movements, interherd contacts, or management-related exposure factors.

### 3.2. Animal-Level Seroprevalence of Brucella Infection and Associated Risk Factors Based on cELISA

The individual animal-level seroprevalence of *Brucella* infection among camels in the Abu Dhabi Emirate was estimated to be 5.9% (95% CI: 3.7–8.9), as determined by cELISA testing (Table 3).

Multivariate binary logistic regression analysis revealed significant geographic and management-related risk factors. Camels located in the Abu Dhabi and Eastern regions had 22.9 (95% CI: 2.07–254.05) and 12.38 (95% CI: 1.43–107.27) times higher odds of being seropositive, respectively, than those located in the Al Dhafra region (Table 4). Moreover, camels managed under farm and regular izba systems were 8.55 (95% CI: 1.23–107.10) and 11.52 (95% CI: 2.82–47.14) times more likely to test seropositive, respectively, compared to camels in random *Izba*, underscoring the influence of husbandry practices on *Brucella* exposure and transmission risk.

### 3.3. Clinic-Level Seroprevalence of Brucella Infection in Camels Across the Three Regions

**Figure 3:**
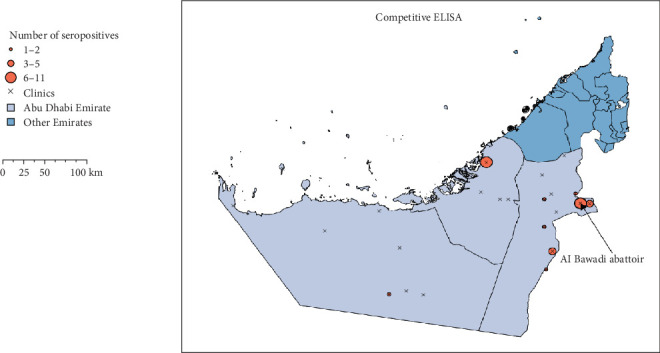
Distribution of seropositive camels by veterinary clinic catchment areas across the three regions of the Emirate of Abu Dhabi. In the Abu Dhabi region, seropositive camels were identified only in the vicinity of Samha Veterinary Clinic. In the Eastern region, positive cases were recorded around the Malaqet and Wagan Veterinary Clinics. In the Al Dhafra region, only a single seropositive camel was detected near the Husaan and Madinat Zayed Clinics.

The distribution of seropositive camels at the veterinary clinic (hospital) and abattoirs is shown in Figure 3. In the Abu Dhabi region, seropositive cases were exclusively detected in camels originating from the areas surrounding the Samha Veterinary Clinic. In the Eastern region, a comparatively higher number of seropositive camels were observed, particularly among animals raised near the Malaqet and Wagan Veterinary Clinics. In contrast, the Al Dhafra region exhibited relatively few seropositive camels, with sporadic cases recorded near the Husaan and Madinat Zayed Clinics.

### 3.4. Seroprevalence of Brucella Infection in Camels Slaughtered at Al Bawadi Abattoir

The seroprevalence of *Brucella* among camels slaughtered at the Al Bawadi abattoir was 3.0% (95% CI: 1.51%–5.30%). Descriptive statistical analysis indicated a significant association between seropositivity and camel age (Fisher's exact test = 6.33; *p*=0.04), as shown in Table 5. However, this association was not supported by the subsequent multivariate logistic regression analysis, suggesting that age alone may not be an independent predictor of infection risk in this population.

### 3.5. Seroprevalence of Brucella Infection Among Abattoir Workers at Al Bawadi Abattoir

The seroprevalence of *Brucella* among abattoir workers in the Al Bawadi abattoir was 8.1% (95% CI: 3.31%–16.0%). Descriptive analysis showed no statistically significant association (*p*  > 0.05) between seropositivity and any of the potential risk factors assessed (Table 6).

### 3.6. Detection of Brucella DNA in Sera of Seropositive Camels and Abattoir Workers

#### 3.6.1. Detection of Brucella Species in Seropositive Camels

**Figure 4:**
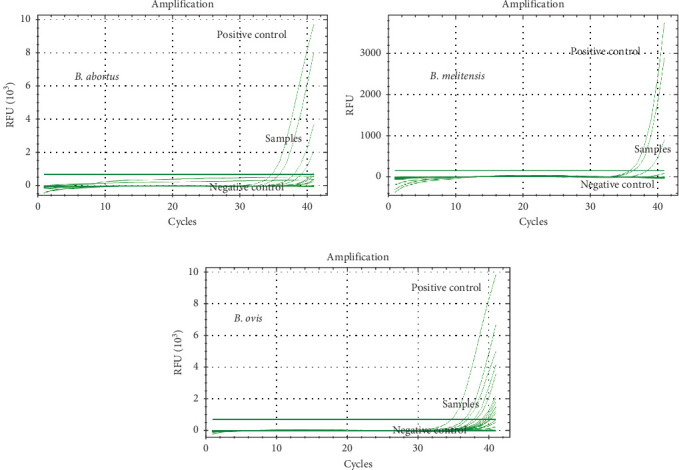
Detection of *Brucella* species DNA in the sera of seropositive camels using qPCR. DNA from three *Brucella* species—*B. ovis*, *B. abortus*, and *B. melitensis*—was identified. *B. ovis* was the most frequently detected species, whereas *B. melitensis* was the least commonly detected.

The presence of DNA from three *Brucella* species (*B. abortus*, *B. melitensis*, and *B. ovis*) was assessed in serum samples from 30 seropositive camels, including 20 field camels and 10 abattoir camels. The results of *Brucella* species identification based on qPCR detection are summarized in Table 7. Overall, 64.5% (20/31) of the camels tested positive for *Brucella* DNA. The DNA of *B. ovis* was the most frequently identified, detected as a single infection in 10 camels and as part of a mixed infection in two additional cases (see Figure 4 and Table 7). *B. abortus* DNA was found in six camels as a single infection and in three others in combination with either *B. melitensis* or *B. ovis*. Similarly, *B. melitensis* was detected in three camels as a single infection and in three additional camels as part of a mixed infection. These findings highlight the cocirculation and coinfection potential of multiple *Brucella* species in the camel populations.

#### 3.6.2. Detection of Brucella Species in Seropositive Abattoir Workers

**Figure 5:**
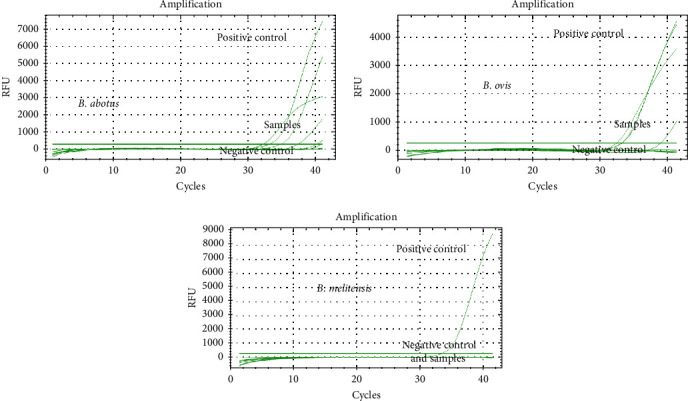
Detection of *Brucella* species DNA in the sera of seropositive abattoir workers using qPCR. *B. abortus* DNA was identified in three individuals, and *B. ovis* DNA was detected in two individuals. *B. melitensis* DNA was not detected in any of the seven seropositive workers.

Sera from seven seropositive abattoir workers were analyzed using qPCR for the presence of *B. abortus*, *B. melitensis*, and *B. ovis* DNA. *B. abortus* DNA was detected in three individuals, whereas *B. ovis* DNA was detected in two individuals (Figure 5 and Table 8). Notably, *B. melitensis* DNA was not detected in any of the samples tested. One individual (ID no. H-42) tested positive for both *B. abortus* and *B. ovis* DNA, indicating a mixed infection.

## 4. Discussion

Camels in the UAE are economically important. As vital livestock species in arid environments, camels contribute to food security through meat and milk production and hold substantial cultural and economic value in the racing and tourism sectors. Brucellosis, a chronic and debilitating zoonosis, undermines these contributions by impairing reproductive performance, decreasing productivity, and restricting trade opportunities. These findings align with those of Dadar and Alamian [17], who demonstrated that *Brucella melitensis* could be isolated from seronegative camels, indicating that reliance on serological assays alone may underestimate true infection burden. Our detection of multiple *Brucella* species in seropositive camels underscores the importance of integrating molecular diagnostics for enhanced surveillance accuracy.

This study assessed the seroprevalence and molecular detection of *Brucella spp*. in camels and abattoir workers in the Emirate of Abu Dhabi. The findings contribute to the growing body of evidence on the One Health implications of camel brucellosis, supporting earlier work that identified occupational exposure risks and diagnostic challenges [17]. The observed seroprevalence aligns with recent estimates from Saudi Arabia and Tunisia, where brucellosis continues to impact camel health and human safety in high-risk occupations [18, 14]. Moreover, our use of molecular diagnostics addresses limitations of prior serological-only surveys and confirms the circulation of multiple *Brucella* species, including *B. ovis*, which had previously been underreported in camels. These insights corroborate findings from the global meta-analysis by Bamaiyi et al. [16], which emphasized the need for improved diagnostic integration and policy alignment across endemic regions. Our findings are also consistent with the global synthesis by Dadar et al. [2], who identified sex, herd size, and geographic location as key risk factors in camel brucellosis. These risk factors were similarly significant in our UAE study, especially in abattoir environments where occupational exposure further increases transmission risks.

The cELISA demonstrated a robust diagnostic capacity. According to the manufacturer (Ingezim *Brucella* Compac 2.0, Ingenasa, Spain), the assay offers 98% sensitivity and 99% specificity. Compared to the complement fixation test (CFT), cELISA is more sensitive, although marginally less specific [25, 26]. cELISA is simpler and more suitable for samples of suboptimal quality [26]. However, the inability to differentiate between infected and vaccinated animals (DIVAs limitation) is a fundamental drawback of serological diagnostics for brucellosis [27]. Fortunately, vaccination is not practiced in camels in the UAE, thus eliminating potential interference. Since cELISA primarily detects IgG, it reflects previous exposure rather than active infection.

The estimated herd-level seroprevalence of *Brucella* in camels is 10.8%, which is significantly associated with herd size. Although similar UAE-based data are lacking for direct comparison, studies from neighboring regions have provided a context. Wernery [8] noted that the seroprevalence may exceed 40% in mixed herds, especially where camels cohabitate with small ruminants. This aligns with our observation that all seropositive herds in the current study kept camels along with sheep and goats.

Herd size has emerged as a risk factor, with medium and large herds showing higher seropositivity, possibly due to increased animal density and contact-facilitating transmission. Similar results were reported in Ethiopian camel herds [28], confirming the influence of management scale on brucellosis risk. At the individual animal level, seroprevalence was 5.9%, categorizing it as low to moderate [29]. Recently, overall seroprevalences of 1.7% and 5.8% were reported in different livestock species including in camels, in the Emirate of Abu Dhabi by serial and parallel testing, respectively [30], which is similar to the results in this study. Earlier studies in the eastern region of Abu Dhabi have indicated variable prevalence levels: 2.0% in breeding camels, 6.0% in racing camels [31], 0.2% in other samples [8], and 1.5% in racing animals. Data from 1991 to 1996 revealed a decline from 5.8% to 0.1% following government control efforts [8]. This historical context supports the current trend of a low prevalence. Regionally, the animal-level prevalence of *Brucella* antibodies in camels ranges from 0% to 30%, with higher rates observed in Egypt, Sudan, and Qatar [13, 14, 32–34].

Molecular testing using qPCR revealed *Brucella* DNA in 67% of the tested seropositive camels. Interestingly, *B. ovis* was the most frequently detected species, both alone and in mixed infections. This contrasts with previous studies that highlighted *B. abortus* and *B. melitensis* as being dominant in camels [11, 35–37]. Only one prior report from Tunisia [38] has identified *B. ovis* in camels. As camels are spillover hosts, infection patterns reflect interspecies contact. Given that *B. ovis* primarily infects sheep [39] and that sheep–camel corearing is common in the UAE, spillover from infected sheep likely explains this pattern [40].

The detection of *B. abortus* (six cases) and *B. melitensis* (three cases) was less frequent. The low detection rate of the latter may correlate with its limited circulation in small ruminants in the region, as confirmed in parallel studies [40]. Although *B. ovis* is less pathogenic to humans, its presence in camels raises concerns regarding livestock productivity and diagnostic complexity.

Among abattoir workers, five of seven seropositive individuals were qPCR-positive—three for *B. abortus* and two for *B. ovis*. One worker had DNA from both species. None of the samples tested positive for *B. melitensis*. This aligns with the absence of *B. melitensis* DNA in camels and supports the hypothesis that *B. ovis* is transmitted from sheep via occupational exposure.

Human brucellosis remains a concern in the UAE. Between 2010 and 2015, 480 confirmed human cases were reported, with an incidence of 3.3 per 100,000 people, increasing to 10 per 100,000 in male expatriates working in small livestock operations [40]. Historical outbreaks in 1996 included *B. melitensis* biovar 2 [41]. Additionally, a study at Tawam Hospital reported 91 pediatric brucellosis cases between 2009 and 2017, mostly associated with unpasteurized camel milk [42]. Neurobrucellosis has also been reported [42]. These findings underscore the zoonotic risk of *Brucella spp*., particularly in the occupational and dietary exposure contexts.

In summary, this study confirmed the presence of *Brucella* infection in camels and abattoir workers in the Emirate of Abu Dhabi, with a high prevalence of *B. ovis*. These findings advocate for enhanced surveillance, molecular diagnostics, and awareness campaigns to control this neglected zoonosis in both animals and humans.

## 5. Conclusion

The detection of *Brucella* species in both camels and abattoir workers underscores the dual threat posed by brucellosis to livestock productivity and public health in the UAE camel meat value chain. These findings highlight the need for an integrated One Health approach that combines veterinary, public health, and food safety interventions. Strengthening surveillance, promoting biosecurity, and implementing targeted control measures are essential to reducing economic losses in the livestock sector and safeguarding the health of those at the animal–human interface. In accordance with the recommendations of the FAO, the World Health Organization (WHO), and the WOAH will be essential to mitigate the transmission of *Brucella* spp. among livestock and reduce the risk of human infection. Sustained commitment to intersectoral collaboration and the One Health approach will be key to managing and ultimately eliminating brucellosis in endemic areas.

## Figures and Tables

**Table 1:** The primers used for the amplification of the DNA of *Brucella* genus and species.

*Brucella* spp.	Target gene	Forward primer	Reverse primer
*B. abortus*	*alkB*	5′-GCGGCTTTTCTATCACGGTATTC-3′	5′-CATGCGCTATGATCTGGTTACG-3′
*B. ovis*	*aroA*	5′-CGACCACCGCATCGC-3′	5′-CCGGCTTTTCCGATGCAA-3′
*B. melitensis*	*BMEI1162*	5′-ACAAGCGGCACCCCTAAAA-3′	5′-CATGCGCTATGATCTGGTTACG-3′
Positive control	GAPDH	5′-CCACCCATGGCAAATTCC-3′	5′-TCGCTCCTGGAAGATGGTG-3′

Abbreviation: GAPDH, Glyceraldehyde 3-phosphate dehydrogenase.

**Table 2:** Competitive ELISA-based seroprevalence of *Brucella* infection in the herds of camels in the Emirate of Abu Dhabi.

Risk factor	Category	Total herd	Positive herd	Percent (95% CI)	*χ* ^2^	*p*-Value
Region	Abu Dhabi	31	1	3.2 (0.1, 16.7)	3.878^a^	0.119
	Eastern	52	6	11.5 (4.4, 23.4)	—	—
	Al Dhafra	19	4	21.1 (6.1, 45.6)	—	—

Holding type	Farm	11	0	0.0 (0.0, 28.5)	2.396^a^	0.307
	Regular *izbas*	59	9	15.3 (7.2, 27.0)	—	—
	Random *izbas*	32	2	6.3 (0.8, 20.8)	—	—

Herd size	Small (≤20)	80	5	6.3 (0.8, 20.8)	7.726^a^	0.015
	Medium (20–50)	18	5	27.8 (9.7, 53.5)	—	—
	Large (≥50)	4	1	25.0 (0.6, 80.6)	—	—

Mixed with camels	No	5	0	0.0 (0.0, 41.1)	—	0.438
	Yes	97	11	11.3 (5.8, 19.4)	—	—

Total		102	11	10.8 (5.5, 18.5)	—	—

^a^Fischer exact test

**Table 3:** Competitive ELISA-based association of animal seroprevalence of *Brucella* infection in camels with different risk factors on the basis univariate binary logistic regression analysis.

Risk factor		cELISA		Total	Prevalence (%)	95% CI	Odd ratio (95% CI)	*χ* ^2^	*p*-Value
		Negative	Positive						
Region	Abu Dhabi	68	10	78	12.82	6.3, 22.3	16.03 (2.01, 128.03)	11.737^a^	0.002
	Eastern	158	10	168	5.95	2.9, 10.7	6.90 (0.87, 54.68)	—	—
	Al Dhafra	109	1	110	0.91	0.0, 45.0	1	—	—

Type of holding	—	—	—	—	11.11	1.4, 34.7	5.17 (0.97, 27.67)	18.535	<0.0001
	Farm	16	2	18	—	—	—	^a^	—
	Regular izba	71	13	84	15.48	8.5, 25.0	7.57 (2.78, 20.63)	—	—
	Random izba	248	6	254	2.36	0.8, 5.1	1	—	—

Age	Young	17	1	18	5.56	0.1, 27.3	1	^a^	0.999
	Adult	318	20	338	5.92	3.7, 9.0	1.07 (0.14, 8.45)	—	—

Body condition	Good	7	0	7	—	0, 41.0	0	0.515^a^	0.508
	Moderate	76	4	80	5.00	1.4, 12.3	0.78 (0.25, 2.39)	—	—
	Poor	252	17	269	6.32	3.7, 9.9	1	—	—

Reproductive status	Dry	129	11	140	7.86	4.0, 13.6	1.49 (0.32, 7.05)	8.649^a^	0.027
	Pregnant	80	0	80	—	0, 4.5	0	—	—
	Lactating	91	8	99	8.08	3.6, 15.3	1.54 (0.31, 7.60)	—	—
	Young	35	2	37	5.41	0.7, 18.2	1	—	—

Abortion	Yes	6	0	6	—	0, 45.9	0	^a^	0.999
	No	305	21	326	6.44	4.0, 9.7	1	—	—

Origin	Born in	309	21	330	6.36	4.0, 9.6	1	^a^	0.382
	Purchased	25	0	25	—	0, 13.7	0	—	—

Total		335	21	356	5.90	3.7, 8.9	—	—	—

^a^Fischer exact test.

**Table 4:** Competitive ELISA-based association of animal seroprevalence of *Brucella* infection in camels with different risk factors on the basis of multivariable binary logistic regression analysis.

Risk factor	Category	Odd ratio (95% CI)	Wald test	*p*-Value
Region	—	—	6.609	0.037
	Abu Dhabi	22.90 (2.07, 254.05)	6.506	0.011
	Eastern	12.38 (1.43, 107.27)	5.212	0.022
	Al Dhafra	1	—	—

Type of holding	—	—	12.225	0.002
	Farm	8.55 (1.23, 107)	4.686	0.03
	Regular *izbas*	11.52 (2.82, 47.14)	11.566	0.001
	Random *izbas*	1	—	—

Reproductive status	—	—	6.884	0.076
	Dry	0.13 (0.02, 1.04)	3.699	0.054
	Pregnant	0	0	0.996
	Lactating	0.96 (0.16, 5.61)	0.002	0.963
	Young	1	—	—

**Table 5:** Competitive ELISA-based association of seroprevalence of *Brucella* infection with camel-related rink factors analyzed by multivariable logistic regression.

Variable	Category	ELISA result		*χ* ^2^	*p*-Value	Odds ratio (OR)	OR (95% CI)	
		Total	Positive (percent)				Lower	Upper
Sex	Female	276	7 (2.54)	0.78^a^	0.38	0.97	0.25	3.73
	Male	92	4 (4.35)	—	—	1	—	—

Age (years)	≤3	111	7 (6.31)	6.33^a^	0.04	0.40	0.09	1.69
	>3 and ≤6	145	3 (2.2)	—	—	0.16	0.02	1.41
	>6	112	1 (1.0)	—	—	1	—	—

Body condition	Poor	24	0 (0.0)	2.57^a^	0.23	0.89	0.000	—
	Medium	44	0 (0.0)	—	—	37829197.23	0.000	—
	Good	300	11 (3.7)	—	—	1	—	—

Total		368	11 (3.0)	—	—	—	—	—

^a^Ficher exact test.

**Table 6:** Competitive ELISA-based association of seroprevalence of *Brucella* infection with potential risk factors in abattoir workers at the Al Bawadi abattoir.

Variables	Categories of variables	ELISA result		Total	*χ* ^2^	*p*-Value
		Negative	Positive (percent)			
Age (years)	≤30	23	4 (4.7)	27	2.47^a^	0.29
	>30 and <40	30	2 (2.3)	32	—	—
	≥40	26	1 (1.2)	27	—	—

Job type	Butcher	65	6 (7.0)	71	0.05^a^	0.05
	Others^a^	14	1 (1.2)	15	—	—

Duration on job (years)	≤5	19	1 (1.2)	20	1.48^a^	0.48
	>5 and ≤10	27	4 (4.7)	31	—	—
	>10	33	2 (2.3)	35	—	—

Country of origin	Bangladesh	19	1 (1.2)	20	1.42^a^	0.84
	Egyptian	10	1 (1.2)	11	—	—
	Other African countries	8	0 (0.0)	8	—	—
	Other Asian countries	10	1 (1.2)	11	—	—
	Pakistan	32	4 (4.7)	36	—	—

Knowledge of Brucellosis	Yes	62	5 (5.8)	67	0.19^a^	0.67
	No	17	2 (2.3)	19	—	—

Previous *Brucella* infection	Yes	6	0 (0.0)	6	0.57^a^	0.45
	No	73	7 (8.1)	80	—	—

Total		79	7 (8.1)	86	—	—

^a^Fischer exact test.

**Table 7:** *Brucella* species detected in the sera of 30 seropositive camels in the Emirate of Abu Dhabi.

Camel ID	*B. ovis* (Ct value)	*B. abortus* (Ct value)	*B. melitensis* (Ct value)	Final result
*Brucella* isolated from the sera of positive camels in the field				
C-188	Negative	Negative	Negative	Negative
C-394	39.76	Negative	Negative	*B. ovis*
C-656	40.0	Negative	Negative	*B. ovis*
C-681	Negative	Negative	Negative	Negative
C-683	Negative	Negative	Negative	Negative
C-684	Negative	Negative	Negative	Negative
C-1345	38.97	Negative	Negative	*B. ovis*
C-1354	37.83	35.87	Negative	*B. ovis and B. abortus*
C-1355	Negative	Negative	Negative	Negative
C-1356	37.0	Negative	Negative	*B. ovis*
C-1357	Negative	Negative	35.78	*B. melitensis*
C-1358	39.18	Negative	Negative	*B. ovis*
C-1359	Negative	40.00	Negative	*B. abortus*
C-1360	36.47	Negative	Negative	*B. ovis*
C-1361	38.20	39.10	Negative	*B. ovis and B. abortus*
C-1363	Negative	Negative	Negative	Negative
C-1872	Negative	38.01	38.34	*B. abortus and B. melitensis*
C-2722	39.71	Negative	Negative	*B. ovis*
C-2856	Negative	Negative	Negative	Negative
C-3132	39.71	Negative	Negative	*B. ovis*
Negative template control	Negative	Negative	Negative	—
Positive control	34.71	34.70	36.25	—
*Brucella* species isolated from camels slaughtered at camels				
C-115	Negative	Negative	Negative	Negative
C-117	40.00	Negative	Negative	*B. ovis*
C-256	Negative	39.81	Negative	*B. abortus*
C-271	Negative	39.01	Negative	*B. abortus*
C-272	Negative	Negative	Negative	Negative
C-273	Negative	Negative	36.36	*B. melitensis*
C-283	Negative	39.07	Negative	*B. abortus*
C-287	Negative	39.07	Negative	*B. abortus*
C-290	Negative	38.46	Negative	*B. abortus*
C-298	35.60	Negative	Negative	*B. ovis*
C-302	Negative	Negative	Negative	Negative
Negative template control	Negative	Negative	Negative	—
Positive control	32.82	31.33	33.39	—

**Table 8:** *Brucella* species detected in the sera of seven seropositive abattoir workers in the Emirate of Abu Dhabi.

Sample ID (abattoir workers)	*B. ovis* (Ct value)	*B. abortus* (Ct value)	*B. melitensis* (Ct value)	Result
H-18	Negative	34.88	Negative	*B. abortus*
H-20	Negative	37.37	Negative	*B. abortus*
H-41	Negative	40.00	Negative	*B. abortus*
H-42	31.45	32.24	Negative	*B. abortus* and *B. ovis*
H-45	Negative	Negative	Negative	Negative
H-72	32.12	Negative	Negative	*B. ovis*
H-86	38.05	Negative	Negative	*B. ovis*
GAPDH (positive control)	32.08	33.20	32.06	—
Negative template control	Negative	Negative	Negative	—

## Data Availability

The data can be obtained from the authors.
